# Optimization of wavelet coherence analysis as a measure of neural synchrony during hyperscanning using functional near-infrared spectroscopy

**DOI:** 10.1117/1.NPh.7.1.015010

**Published:** 2020-02-28

**Authors:** Xian Zhang, J. Adam Noah, Swethasri Dravida, Joy Hirsch

**Affiliations:** a Yale School of Medicine, Brain Function Laboratory, Department of Psychiatry, New Haven, Connecticut, United States; b Yale School of Medicine, Interdepartmental Neuroscience Program, New Haven, Connecticut, United States; c Yale School of Medicine, Medical Scientist Training Program, New Haven, Connecticut, United States; d Yale School of Medicine, Department of Neuroscience, New Haven, Connecticut, United States; e Yale School of Medicine, Department of Comparative Medicine, New Haven, Connecticut, United States; f University College London, Department of Medical Physics and Biomedical Engineering, London, United Kingdom

**Keywords:** dynamic neural coupling, wavelet coherence analysis, functional near-infrared spectroscopy, hyperscanning, cross-brain coherence, neural synchrony, social neuroscience

## Abstract

**Significance:** The expanding field of human social interaction is enabled by functional near-infrared spectroscopy (fNIRS) that acquires hemodynamic signals during live two-person interactions. These advances call for development of methods to quantify interactive processes.

**Aim:** Wavelet coherence analysis has been applied to cross-brain neural coupling. However, fNIRS-specific computations have not been explored. This investigation determines the effects of global mean removal, wavelet equation, and choice of oxyhemoglobin versus deoxyhemoglobin signals.

**Approach:** We compare signals with a known coherence with acquired signals to determine optimal computational approaches. The known coherence was calculated using three visual stimulation sequences of a contrast-reversing checkerboard convolved with the canonical hemodynamic response function. This standard was compared with acquired human fNIRS responses within visual cortex using the same sequences.

**Results**: Observed coherence was consistent with known coherence with highest correlations within the wavelength range between 10 and 20 s. Removal of the global mean improved the correlation irrespective of the specific equation for wavelet coherence, and the oxyhemoglobin signal was associated with a marginal correlation advantage.

**Conclusions**: These findings provide both methodological and computational guidance that enhances the validity and interpretability of wavelet coherence analysis for fNIRS signals acquired during live social interactions.

## 1 Introduction

Emerging theoretical frameworks in neuroscience focus on interpersonal interactions and the challenge to understand communicating brains. Within the context of this “neuroscience of two,” the human dyad is considered the functional unit and focuses an investigational spotlight on questions related to how two brains work together to achieve “wireless” communications from one brain to the other. Human brain processes and organization are conventionally studied using functional magnetic resonance imaging (fMRI), where subjects are studied one at a time in noninteractive conditions due to the constraints of the imaging technology. These constraints include lying in a confined scanning tunnel with restrictions on head movements, a ban on speech production due to the limited tolerance of head movement, and obstacles related to hearing due to the loudness of the machine noise related in part to the rapid switching of the gradients. Nonetheless, the technique of hyperscanning was pioneered using fMRI by chaining together two scanners and setting up conditions with limited interactions between participants.^1^ The research goal was to interrogate neural systems engaged during the processing of spontaneous and reciprocal social interactive cues. However, this technology does not permit imaging within natural conditions where two individuals share information in real time, including face-to-face interactions and spoken communications. On the other hand, recent developments of hyperscanning using functional near-infrared spectroscopy (fNIRS) provide an experimental environment absent a high magnetic field and isolated conditions, which permits neuroimaging in natural and real-time situations. Hyperscanning with fNIRS is a rapidly advancing field focused on pivotal neural topics for investigation including eye-to-eye contact, dynamic facial expressions, and responsive gestures that occur spontaneously in real-time communications. The neurophysiology that underlies interpersonal communication and dynamic interactions between humans has emerged as an active neuroscience research topic opening many new areas of investigation including competition and cooperation, coordination of movements, group musical performances, mother-child interactions, joint decision-making and attention, theory of mind, spoken dialogue, and group interactions.

A proposed theoretical framework for these cross-brain systems is based on temporally synchronous signals that are assumed to reflect shared processes between two brains.^1^ The investigation of neural synchrony and the neural mechanisms that process nuanced social behavior is enabled by advances in hyperscanning (simultaneous imaging of two individuals) using fNIRS techniques where hemodynamic brain responses are acquired in natural conditions. Social cues such as eye contact and facial expressions between individuals occur sporadically and on multiple time scales outside the time frame of conventional “block” experimental paradigms. Computational methodologies based on controlled stimulus events such as those presented in task-based designs are challenged by these spontaneous events. As a consequence, both acquisition techniques and computational methods are currently under development in order to take advantage of the investigative opportunities embedded within two-person neuroimaging paradigms to measure live interactive processes. Here we focus on a computational method to investigate interactive effects that are measured by cross-brain neural coupling.

Wavelet approaches decompose complex waveforms into signals with various temporal periods. As such, wavelets have been proposed for revealing coupled neural processes between interacting dyads where shared social signals are transient and spontaneous. In particular, cross-brain neural synchrony measured with wavelet coherence analysis has been applied to investigate interactive behaviors, such as cooperative and competitive gameplay,^2^^–^^4^ synchronized finger tapping,^5^ unstructured conversation,^6^ dyadic singing and humming,^7^ button-pressing,^8^ creative problem solving,^9^ face-to-face interaction,^10^ structured talking and listening,^11^ playing poker against a human or computer opponent,^12^ judging intentions and fairness in economic exchanges,^13^ and following and leading.^14^^,^^15^ Although the wavelet coherence computations have been applied previously in these and other applications, the computational factors that affect the power of the analysis have not been explored for fNIRS signals. Here we use a method of actual acquired signals with a known wavelet coherence in order to determine optimal approaches for wavelet coherence analysis applied specifically to fNIRS data.

## 2 Materials and Methods

### 2.1 Participants

Fifteen healthy adult participants were included in this study: mean age=27±8, 11% female. All participants provided written informed consent in accordance with guidelines approved by the Yale University Human Investigation Committee (HIC #1501015178) and were reimbursed for their participation.

### 2.2 Stimulus and Predicted Signal Coherence

**Fig. 1:**
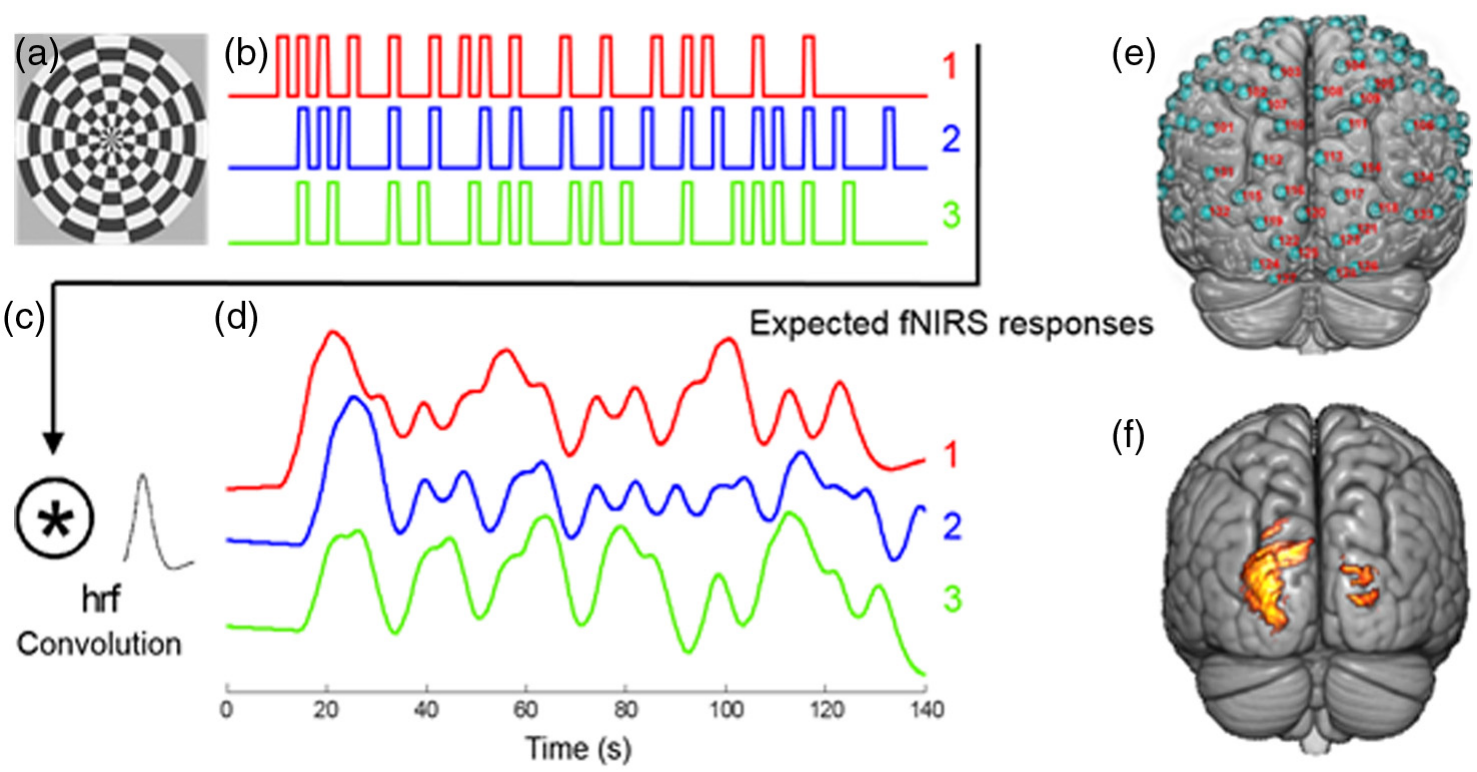
(a) The reversing checkerboard stimulus pattern subtended 15 degrees of visual angle. (b) The three stimulation sequences: 1, 2, and 3. Each vertical bar represents a two-second event during which the rate of reversal was 200 ms. The checkerboard was stationary during the interevent intervals. Approximately 16 events occurred during a 2-min run for all sequences. (c) The hemodynamic response function was convolved with each stimulation sequence. (d) The expected fNIRS responses for each sequence. (e) The channels (green circles) that cover the posterior part of the brain (occipital lobe) are identified by the red numbers and represent locations of detected hemodynamic signals. (f) The group analysis for 15 subjects combining all the sequences and both oxy- and deoxyhemoglobin spatially filtered signals are indicated by the clusters on the rendered brain (p<0.05).

A reversing checkerboard visual stimulus that subtended ∼15  deg of visual angle on the retina of the viewer [Fig. 1(a)] was used to generate responses in the visual cortex. Each stimulus event lasted two seconds, and the checkerboard reversed black and white polarity every 200 ms. This stimulation paradigm was designed to simulate random and brief events similar to the perceptual experience of detecting a rapid series of social cues during live interactions between dyads. Three random sequences were presented for 2 min each [Fig. 1(b)] and repeated twice. Convolution of the random sequences with the hemodynamic response function [Fig. 1(c)] revealed the expected fNIRS/neural responses [Fig. 1(d)]. Subjects were recorded separately and computationally paired during analyses. By exhaustive pairing with all subjects, the 15 subjects provided 210 pairs of fNIRS responses. The three nonidentical sequences were designed so that the expected wavelet coherence for a “1–2” pair [red and blue in Fig. 1(d), mean coherence = 0.57] was consistently greater than that for a “1–3” pair [red and green in Fig. 1(d), mean coherence = 0.27] over a wide range of wavelet components.

### 2.3 Signal Acquisition

Hemodynamic signals were acquired for all participants using an 80-fiber continuous-wave fNIRS system (LABNIRS, Shimadzu Corp., Kyoto, Japan). The temporal resolution for signal acquisition was 123 ms. In the LABNIRS system, three wavelengths of light (780, 805, and 830 nm) are delivered by each emitter, and each detector measures the absorbance for these wavelengths. Using the three wavelengths together and a modified Beer–Lambert equation, absorption was converted to concentration changes for deoxyhemoglobin and oxyhemoglobin.^16^

Note: These wavelengths of light are not to be confused with the wavelength units applied to wavelet coherence analysis.

### 2.4 Optode Localization

The optode layout provided full-head coverage, including 134 channels with a spatial resolution of ∼3  cm. The channels that cover bilateral visual cortex are shown in Fig. 1(e), indicated by red labels. Signals within this region are reported. Anatomical locations of optodes were determined for each participant in relation to standard head landmarks [inion, nasion, top center (Cz), and left and right tragi] using a Patriot 3D Digitizer (Polhemus, Colchester, Vermont). Montreal Neurological Institute (MNI) coordinates for the channels were obtained using NIRS-SPM^17^ with MATLAB (Mathworks, Natick, Massachusetts).

### 2.5 Signal Processing

Baseline drift was removed using wavelet detrending (NIRS-SPM). Systemic global effects (e.g., blood pressure, respiration, and blood flow variation) have previously been shown to alter relative blood hemoglobin concentrations^18^^–^^20^ and present a possible confound of inadvertently processing hemodynamic responses that are due to systemic effects rather than neurovascular coupling.^21^ These global components were removed using a principle component analysis spatial filter.^22^^,^^23^ This technique exploits advantages of distributed optode coverage by spatial filtering to distinguish signal components originating from local sources (assumed to be specific to neural events under investigation) from global components assumed to be systemic factors that originate from non-neural sources. Findings are similar for both spatially filtered OxyHb and deOxyHb signals, as illustrated in Fig. 6.

First-level general linear model analysis was performed on the oxyhemoglobin, deoxyhemoglobin, and combined signal from the occipital lobe of each participant. Inclusion in the analysis required that t-values for the separate oxyhemoglobin and deoxyhemoglobin signals showed brain activity at t=2.5 (p≤0.001). For each subject, any channel that met these criteria was included in the analysis. Since both oxyhemoglobin and deoxyhemoglobin signals showed robust responses to visual stimuli, we summed the neural activity conveyed in both signals by reversing the polarity of the deoxyhemoglobin signal and taking the sum of the two spatially filtered signals. This is referred to as the combined signal. Figure 1(f) shows the group visual activity using SPM second-level analysis.

### 2.6 Choice of Wave Function Used in Wavelet Coherence Analysis

**Fig. 2:**
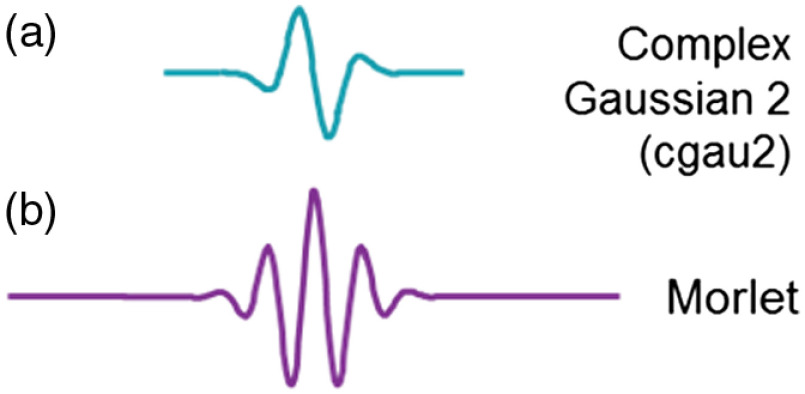
The waveforms of both wave functions: (a) complex Gaussian 2 (cgau2) and (b) Morlet (bottom).

Wavelet analysis involves the choice of wavelet functions. The optimal wave function is expected to match the waveform of the underlying signal. In this paper, we used the Complex Gaussian 2 (“cgau2” from the MATLAB wavelet toolbox) wave function based on its proximity to the hemodynamic response function. Figure 2 shows the cgau2 wave function used in this paper and the commonly used Morlet function. The preferred choice of a wavelet function is a match to the targeted signal. The alternative Morlet function contains multiple cycles and thus is optimal for detecting high-frequency signals such as the beta and gamma waves in EEG. However, for fNIRS data, such multicycle signals rarely occur, especially for the signal of wavelengths around 10 to 20 s. In comparison, the cgau2 wave function is closer to the waveform of a typical fNIRS response.

### 2.7 Averaging Wavelet Coherence Along the Time Domain

**Fig. 3:**
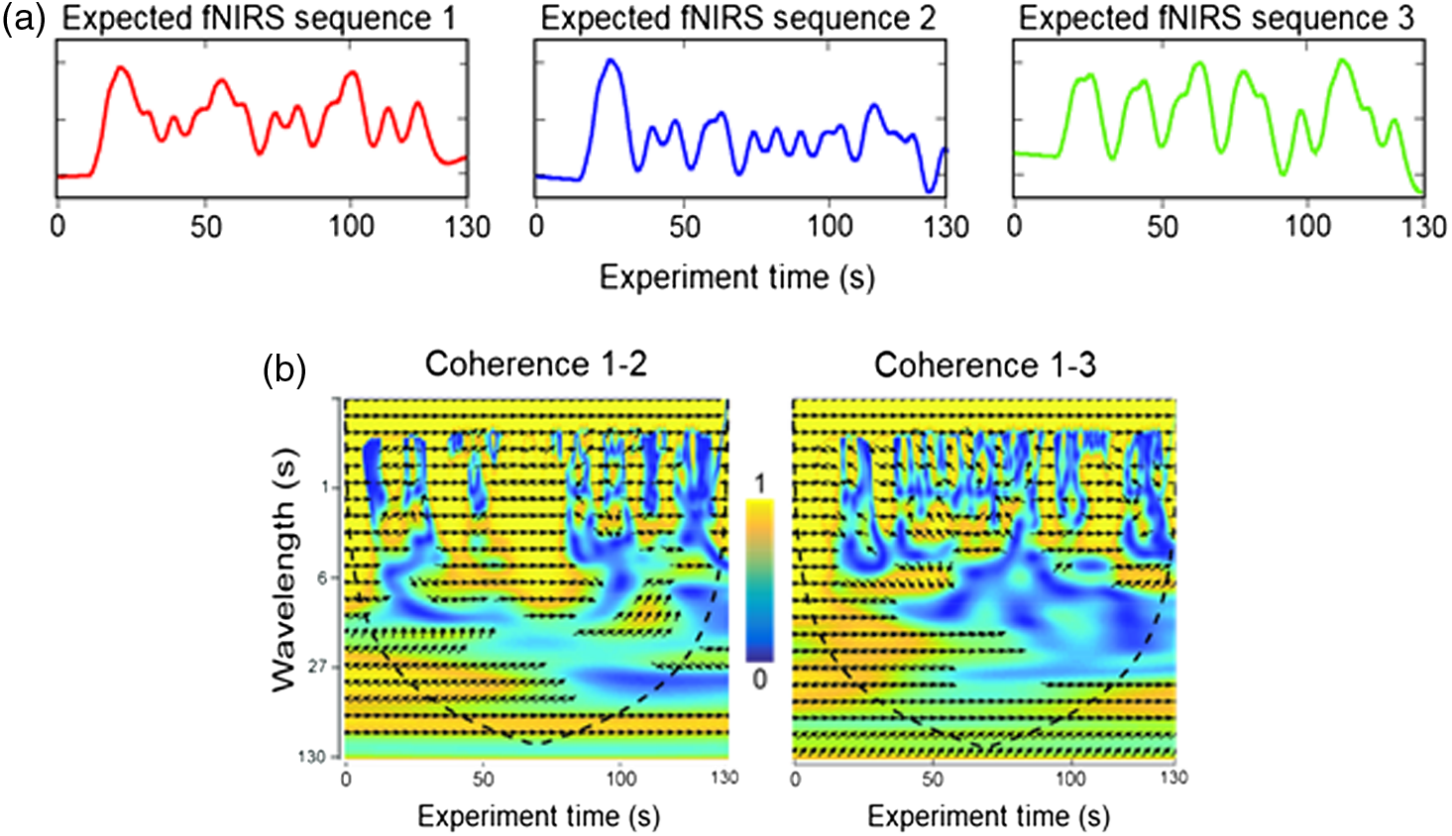
(a) The expected fNIRS paired responses for sequences 1, 2, and 3 [see Fig. 1(d)]. (b) The wavelet coherence matrix derived from 1–2 pairs (left) and 1–3 pairs (right) of expected fNIRS responses. Yellow indicates strong coherence and blue indicates weak coherence. Arrows indicate the relative phase between the two signals. The wavelet coherence for each time point is calculated using data around that time point. At both ends of a record, the coherence has to be calculated with data either before the first sample or after the last sample, which are padded with zeros and are meaningless. The dashed line cone represents the boundary between where coherence values are valid or not.

We measure coherence between fNIRS signals from all pairs of subjects and compare with the expected coherence obtained by convolving the stimulus sequences with the hemodynamic response function [Fig. 3(a)]. Coherence analysis^24^ [Fig. 3(b)] between signal pairs was performed using the MATLAB wavelet toolbox. The result of the wavelet coherence analysis is a two-dimensional complex matrix specified by both time and frequency or wavelength. Each value is a complex number: a+b×i, where the coherence is $\sqrt[{2}]{a^{2}+b^{2}}$ [yellow is high and blue is low in Fig. 3(b)], and the relative phase (related to latency) between two signals is ${\text{tangent}}^{-1}(\frac{b}{a})$ [represented as arrows in Fig. 3(b)].

In this paper, as well as in previous publications,^10^^–^^12^ coherence data were averaged along the time domain to obtain a measure of average coherence:

$$\text{Average coherence through complex value}=\sqrt[{2}]{\;{\left(\frac{\sum_{i=1}^{n}a_{i}}{n}\right)}^{2}+{\left(\frac{\sum_{i=1}^{n}b_{i}}{n}\right)}^{2}\;},$$ (1)

where n is the total number of acquired samples.

An alternative method is to average the coherence values directly:

$$\text{Average coherence directly}=\frac{\sum_{i=1}^{n}{\text{coherence}}_{i}}{n}.$$ (2)

**Fig. 4:**
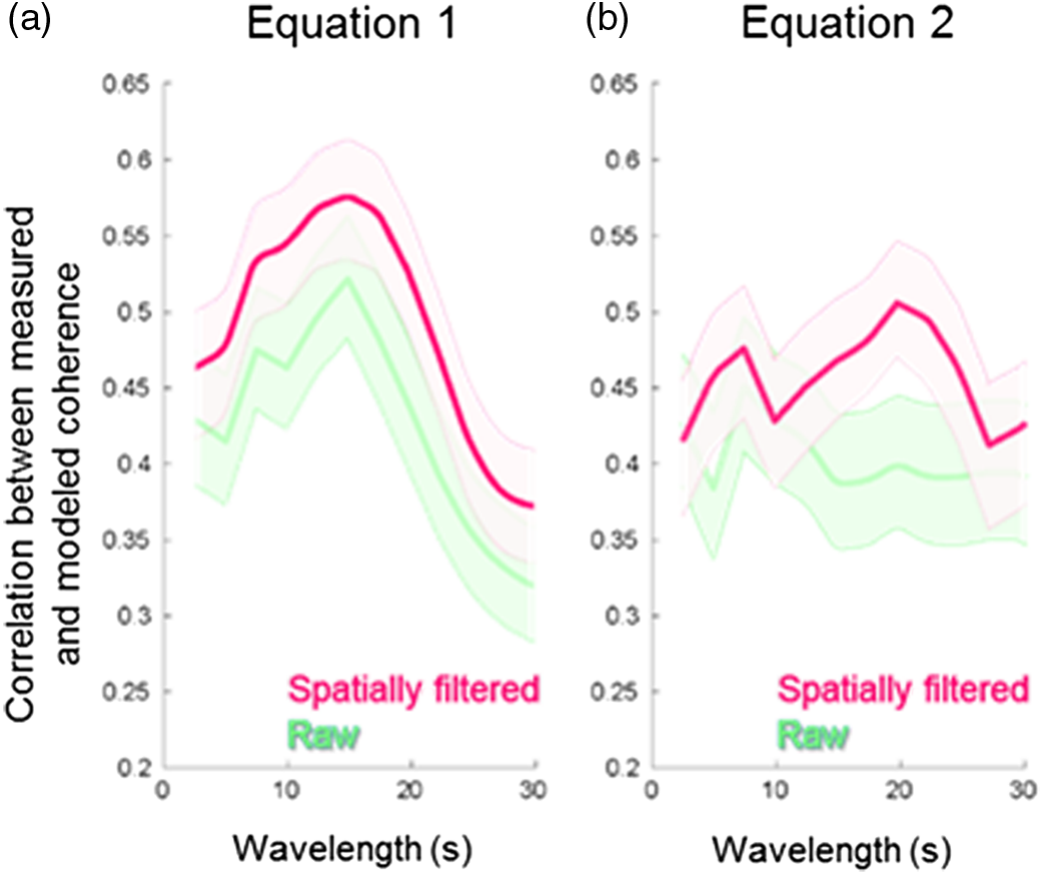
Correlation between the measured coherence from the visual ROI and the expected coherence using the combined fNIRS signals. Red functions show results from signals that have been filtered to remove the global mean. Green functions show results from the “raw,” unprocessed signals. (a) Coherence calculated with Eq. (1) and (b) coherence calculated with Eq. (2).

For example, when coherence values at two time points have opposite phase angles (i.e., $a_{1}=-a_{2}$ and $b_{1}=-b_{2}$), the result of averaging coherence through complex values is 0 [Eq. (1)], and averaging coherence directly results in $\sqrt[{2}]{a_{1}^{2}+b_{1}^{2}}\;\;$[Eq. (2)]. Although Eq. (2) appears straightforward, Eq. (1) imposes more constraints to the latency difference between two signals and is considered to be mathematically more rigorous. Comparison of the two approaches is presented in Fig. 4 (Sec. 3).

**Fig. 5:**
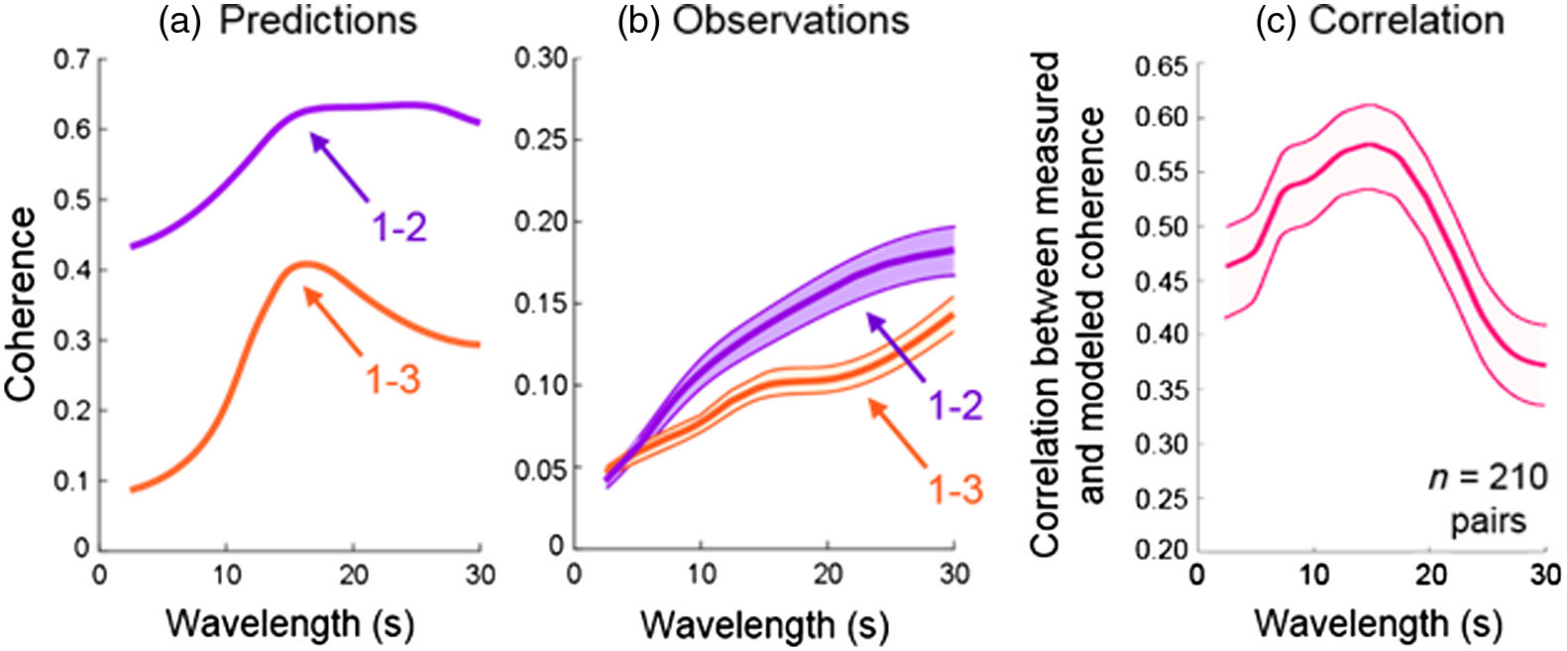
(a) The predicted wavelength-coherence plot between sequences 1 and 2 (purple) and sequences 1 and 3 (orange) obtained by averaging coherence of sequences convolved with the hemodynamic response function along the time dimension using Eq. (1). (b) Observed coherence plots in the visual ROI for the combined spatially filtered oxy- and deoxyhemoglobin signals obtained from all subject pairs [Eq. (1)]. Purple: average coherence between participants viewing sequences 1 and 2, orange: average coherence between participants viewing sequences 1 and 3. (c). The correlation between the measured coherence and the expected coherence [Eq. (1)].

Findings of this study are represented by the average coherence across the entire run along the time dimension (x axis) in a wavelength-coherence plot [as shown in Sec. 3 Figs. 5(a)–5(b)] for wavelengths <30  s, as this is sufficient to cover the scale of predicted neuronal events.

There are four possible sequence pairings: identical (combination of 1–1, 2–2, or 3–3), 1–2, 1–3, and 2–3, with each number corresponding to a random stimulation sequence found in Fig. 1(b). Thus there are a total of four possible predicted and four possible measured coherence values to evaluate the correlation between them. Functional NIRS responses to identical stimulus sequences would be expected to yield a coherence of 1.0 across all frequencies in the absence of noise. However, the presence of random noise in hemodynamic responses would reduce the observed coherences below this theoretical ideal.

## 3 Results

### 3.1 Comparisons between Predicted and Observed Coherence Values of Stimulus Sequence Pairs

**Fig. 6:**
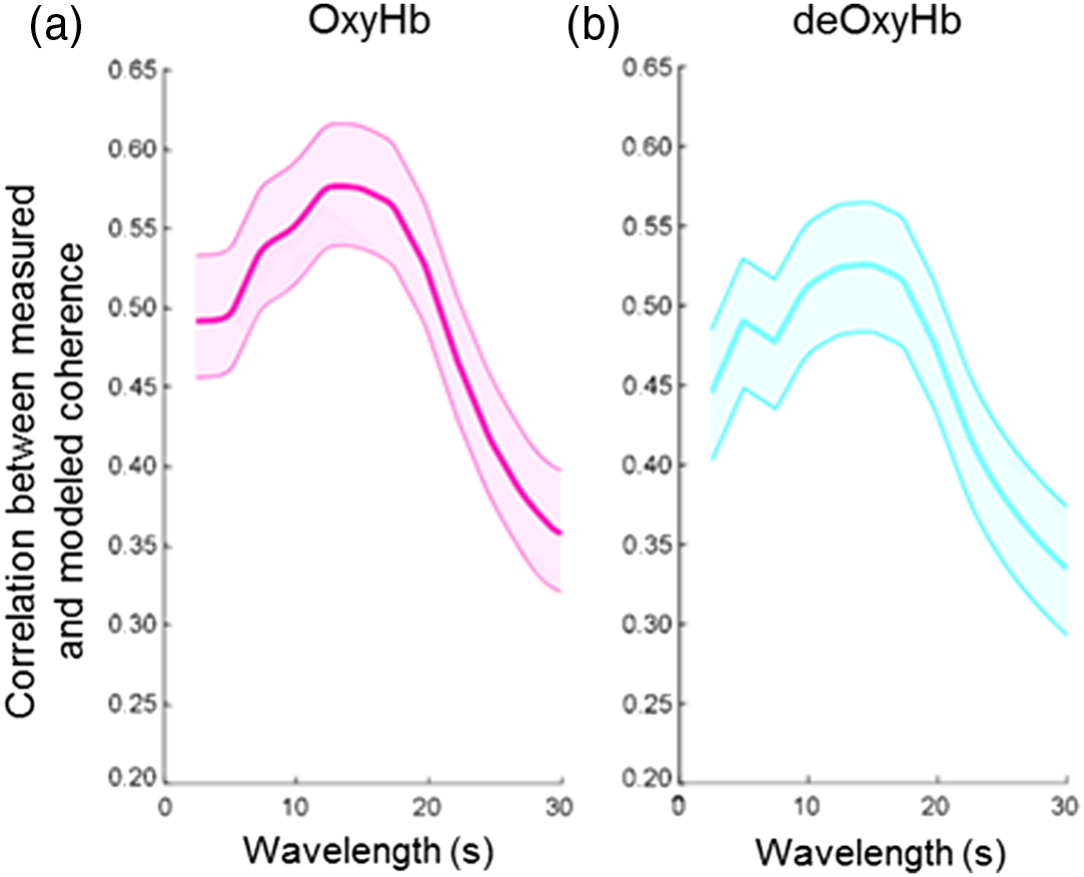
Correlation between measured coherence from the visual ROI and expected coherence using the (a) oxyhemoglobin (OxyHb, magenta) and (b) deoxyhemoglobin (deOxyHb, cyan) fNIRS signals. Coherence values were calculated with Eq. (1).

Two stimulus sequence pairs are shown in Fig. 5(a) (sequences 1 and 2: high-input coherence; sequences 1 and 3: low-input coherence) for each wavelength (x axis) with the predicted fNIRS signal coherence values (y axis). The average observed coherence values for both stimulus sequence pairs (pair 1–2 and pair 1–3) are shown in Fig. 5(b). Note that the relative order of the observed functions [Fig. 5(b)] matches the relative order of the predicted functions [Fig. 5(a)]. However, an upward trend along the wavelength dimension is observed. This phenomenon is related to the noise and the mathematic nature of the wavelet coherence analysis. To generate Fig. 5(b), we averaged the wavelet coherence along the time domain in complex value [Eq. (1)]. For high-frequency signals, there are more independent samples along the time domain. Therefore, the average of random noise will approach zero in complex terms. In contrast, for low-frequency or long-wavelength signals, there are fewer independent samples and thus the average coherence value of random noise will be further away from zero. As a result, Fig. 5(b) shows an upward trend in the wavelet coherence along the wavelength dimension and the apparent high coherence does not indicate stronger signal. The correlation between the predicted and observed coherence shown in Fig. 5(c) presents the true strength of the signal. Figure 5(c) shows the mean (dark line in the middle of shaded standard error area) for correlations between measured and modeled coherence for 210 pairs of subjects over the range of wavelengths. Wavelet coherence measured with fNIRS data best reflect the expected coherence within the wavelength range between ∼10 and 20 s where the correlation between predicted coherence and observed coherence is between 0.5 and 0.6. Such results also hold when using either OxyHb or deOxyHb signals alone (see Fig. 6). These observations are consistent with the conclusion that wavelet coherence analysis based on fNIRS signals provides a measure of neural synchrony between two brains plus noise. The highest correlations extend from ∼8 to 20 s, consistent with the influence of increased noise above and below this range of temporal frequencies. This result suggests that the preferred wavelength of coherence analysis coincides with the characteristics of the hemodynamic response function.

### 3.2 Alternative Equations for Averaging Wavelet Coherence along the Time Domain and Signal Processing.

As shown above, both Eqs. (1) and (2) can be used for calculating the average coherence along the time domain; however, the extent to which the measured coherence reflects the expected coherence differs depending on the equation used. Figure 4 shows the effects of wavelet Eqs. (1) and (2) on the correlations (red functions). The mean (thick lines between thin lines) and standard error (shaded areas) are shown for correlations between the measured and expected coherence calculated with Eq. (1) [Fig. 4(a)] and Eq. (2) [Fig. 4(b)]. Mathematically, Eq. (1) more effectively suppresses false positives by reducing the average value when the relative phase of the two input signals is not consistent along the time domain. Equation (2) accounts for the fact that both partners show signals of the same frequency regardless of the relative latency. The comparison of green and red functions shows the effects of signal processing on the correlations.

Red functions are calculated with spatially filtered signals, and green functions are calculated with “raw” and unprocessed signals and confirm the advantage of spatial filtering.

#### 3.2.1 Effects of signal source

Both oxyhemoglobin signals (left) and deoxyhemoglobin signals (right) can be used for wavelet coherence analysis. Oxyhemoglobin signals have a higher signal-to-noise ratio; thus the correlation between the measured coherence and predicted coherence is slightly higher in this case.

## 4 Discussion and Conclusion

Given the affinity of humans to associate with others, understanding the neural underpinnings of human social behavior is a high-priority scientific objective that is not addressed by conventional computational or experimental methods.^25^^–^^29^ Conventional functional neuroimaging methods are optimized to investigate neural operations in single human brains. However, confinement of the participants and isolation in a supine position required by scanners using magnetic resonance imaging present significant challenges to imaging more than one participant at a time. Although these techniques are sufficient for investigations of neural functions that occur under single subject and noninteractive conditions, these methods rarely interrogate neural systems that are engaged during live two-person social interactions. Nonetheless, it is widely appreciated that human beings are predisposed to interact with each other in natural conditions, and these spontaneous and brief social behaviors represent a large portion of the human behavioral repertoire.

Recent technical advances enable the acquisition of brain signals simultaneously on two people during live and natural interactions and have catalyzed this novel research direction. One such emerging technology is dual-brain fNIRS, where hemodynamic signals are acquired using optical methods and surface-mounted detectors on the head.^30^^,^^31^ Although spatial resolution of fNIRS is limited to ∼3  cm, tolerance to head movement is sufficient for acquisition of valid signals under free-moving and nearly natural conditions. Dual-brain imaging outside of the scanner free of the high magnetic field includes far-reaching opportunities to interrogate human neural processes that underlie natural and upright social behaviors.^32^^,^^33^ Two-brain functional imaging systems also introduce an emerging shift from a single-person theoretical frame of reference to a frame of reference focused on the human dyad. This shift includes computational approaches that model the two-person dyad as a unit. For example, synchrony between signals originating between two brains is assumed to reflect coupled dynamics and has been proposed as a biomarker for sharing socially relevant information.^1^^,^^34^ Observations of neural coupling during interactive tasks have become a cornerstone for an emerging theoretical framework of dynamic cross-brain neural processes.^2^^,^^10^^–^^12^^,^^25^^,^^35^^–^^40^

Although the evidence for the association of cross-brain signal coherence and behavioral synchrony is abundant, the underlying mechanisms for interpersonal behavioral attunement are topics of active investigation. The dyadic frame of reference provides a computational platform for hypothesis testing related to models of behavioral synchrony. This framework has similarities to methods applied to investigations of neural systems within single brains. For example, neural complexes are frequently interrogated by computing psychophysiological interactions (PPI) that are based on correlations between hemodynamic signals originating from remote locations in the brain.^41^ These single-brain functional connectivity computations are performed on residual components of the hemodynamic signal following computational removal of the modeled task. This method assumes that the high-frequency residual oscillations observed in the hemodynamic signals have a neural origin and that their correlations reveal cooperative neural processes. These computational approaches support models where neural linkages between functional systems within single brains form hierarchical neural operations that underlie synchronous complex behaviors. Wavelet analysis of cross-brain hemodynamic signals is an adaptation of the PPI computational methods employed to understand within-brain functional connectivity that extends the approach to cross-brain connectivity. In wavelet analysis, hemodynamic signals are decomposed into their wavelet components,^24^ which effectively removes the low-frequency components while retaining residual (nontask-related) signals.

Findings of this study advance the assumption that cross-brain coherence using wavelet analysis represents neural processes plus measurement error. This is based on the comparison between the predicted neural signal determined by the convolution of the visual stimulus time series and the actual observed neural signal from the visual cortex. Neural activity was predicted by convolution of stimulus sequences with the canonical hemodynamic response function. The order of the observed neural synchrony was consistent with the predictions from the input signals. However, the absolute coherence measures were less than predicted. Stimulus information in this experiment consisted of rapid and varying time sequences and thus simulated the experience of sending and receiving natural social cues such as spontaneous face-to-face and eye-to-eye events shared between interacting dyads. Thus findings are generally consistent with the model of neural coupling where cross-brain coherence is assumed to represent spontaneous and transient shared information between the interacting participants.

Here we confirm that neural coupling as represented by wavelet coherence analysis on hemodynamic signals acquired by fNIRS in response to sequences of visual stimulation reflects underlying neural coherence between two brains. However, the comparison of predicted and observed coherence [Figs. 4 and 5(c)] reveals the inclusion of noise and/or other components. Potential noise sources include error in the fit to the modeled hemodynamic response function, imperfect extraction of systemic and global components in the hemodynamic signal, head motion, and resolution limitations. These factors constrain computational approaches. Nonetheless, overall, these findings validate wavelet analysis as an indicator of neural coupling and as a computational approach for further investigation of the neural mechanisms that underlie behavioral attunement.

Comparison of the levels of coherence and the specific regions that share entrained signals across brains provides a quantitative approach to investigate synchronous processes during social interactions. Social signals are detected by sensory and motor systems including vision, audition, and sensation that are functionally connected to higher level cognitive and executive systems within single brains. It can be expected that further investigations of natural dyadic social processes will show entrainment of cross-brain neural systems that reveal higher-level cognitive and perceptual processes. Findings of this investigation confirm that measures of cross-brain coherence using wavelet analysis contribute toward the development of this theoretical framework.
